# Non-Coding RNA and Frizzled Receptors in Cancer

**DOI:** 10.3389/fmolb.2021.712546

**Published:** 2021-10-04

**Authors:** Alex J. Smith, Kayla M. Sompel, Alamelu Elango, Meredith A. Tennis

**Affiliations:** Division of Pulmonary Sciences and Critical Care Medicine, School of Medicine, University of Colorado, Aurora, CO, United States

**Keywords:** non-coding RNA, cancer, frizzled, Wnt/B-catenin, miRNA, lncRNA, circRNA, chemopreventation

## Abstract

Frizzled receptors have been long recognized for their role in Wnt/β-catenin signaling, a pathway known for its tumorigenic effects. More recent studies of frizzled receptors include efforts to understand non-coding RNA (ncRNA) regulation of these receptors in cancer. It has become increasingly clear that ncRNA molecules are important for regulating the expression of both oncogenic and tumor-suppressive proteins. The three most commonly described ncRNA molecules are microRNAs (miRNAs), long non-coding RNAs (lncRNAs), and circular RNAs (circRNAs). Here, we review ncRNA molecules that directly or indirectly affect frizzled protein expression and downstream signaling. Exploring these interactions highlights the potential of incorporating ncRNA molecules into cancer prevention and therapy strategies that target frizzled receptors. Previous investigations of frizzled receptors and ncRNA have established strong promise for a role in cancer progression, but additional studies are needed to provide the substantial pre-clinical evidence required to translate findings to clinical applications.

## Introduction

Non-coding RNA molecules are known for their role in regulating tumorigenic pathways. Three different non-coding RNA molecules have been identified, including microRNA (miRNA), long non-coding RNA (lncRNA), and circular RNA (circRNA), all of which lack protein coding capacity. MicroRNAs contain complementary nucleotide sequences to specific 5′ or 3′UTR regions of mRNAs, allowing them to bind to the mRNA and inhibit translation (Fan et al., 2019). lncRNAs regulate both at the transcriptional and translational levels by various mechanisms. Research on lncRNA in cancer has focused on the ability of lncRNA to indirectly control signaling pathways by inhibiting transcription of interfering miRNA molecules, or by binding to mature miRNA as a competing endogenous RNA (ceRNA) (Ponting et al., 2009; Li et al., 2020a). CircRNA is an ssRNA molecule formed from the joining of the 5′ end and 3′-poly-A tail during exon splicing events (Memczak et al., 2013). CircRNA manipulates numerous pathways by complementary binding of specific miRNAs, thus inhibiting their activity, similar to lncRNA (Memczak et al., 2013). The ability of circRNAs to significantly enhance or inhibit tumorigenic pathways, along with their increased stability compared to lncRNA, highlights their potential as therapeutic targets for future research (Memczak et al., 2013). There are 10 frizzled (Fzd) protein receptors, categorized as G-protein–coupled receptors (GPCRs) with a seven-span transmembrane domain that activates multiple tumorigenic signaling pathways following wingless type (WNT) ligand binding. Downstream Fzd/Wnt pathways include both the canonical Wnt/β-catenin pathway and the two non-canonical Wnt/PCP and Wnt/Ca^2+^ pathways. The canonical Wnt/β-catenin pathway is a common signaling pathway in cancer where Wnt/Fzd binding inhibits the tertiary GSK3β complex from forming. This inhibition prevents the phosphorylation and inactivation of β-catenin in the cytoplasm. This enables β-catenin to translocate to the nucleus where it associates with the T-cell factor/lymphoid enhancer factor (TCF/LEF) transcription factors to induce the transcription of tumorigenic target genes such as TGF-β (Hendrickx and Leyns, 2008). Meanwhile, the Wnt/PCP and Wnt/Ca^2+^ pathways induce cytoskeletal rearrangements, as well as cell polarity and movement by upregulating intracellular phosphorylation cascades.^5^ Although the role of Fzds in cancer varies by specific Fzd receptor and cancer type, their signaling pathways have emerged as potentially important drug targets due to their critical role in Wnt/β-catenin signaling and cell mobility (Schulte and Kozielewicz, 2020). For instance, compounds that bind to the transmembrane domain of Fzd7 and interfere with Wnt binding could reduce oncogenic signaling in many cancer types (Kalhor et al., 2020). The epilepsy drug carbamazepine binds Fzd8 to suppress β-catenin signaling (Zhao et al., 2020), and Fzd9 may be a critical receptor for a lung cancer chemoprevention drug (Tennis et al., 2010). Identifying non-coding RNAs that enhance or inhibit downstream Fzd signaling will support new approaches to cancer prevention and treatment. Here, we provide a brief review of non-coding RNA associated with Fzd receptors in cancer.

## MicroRNA

**FIGURE 1:**
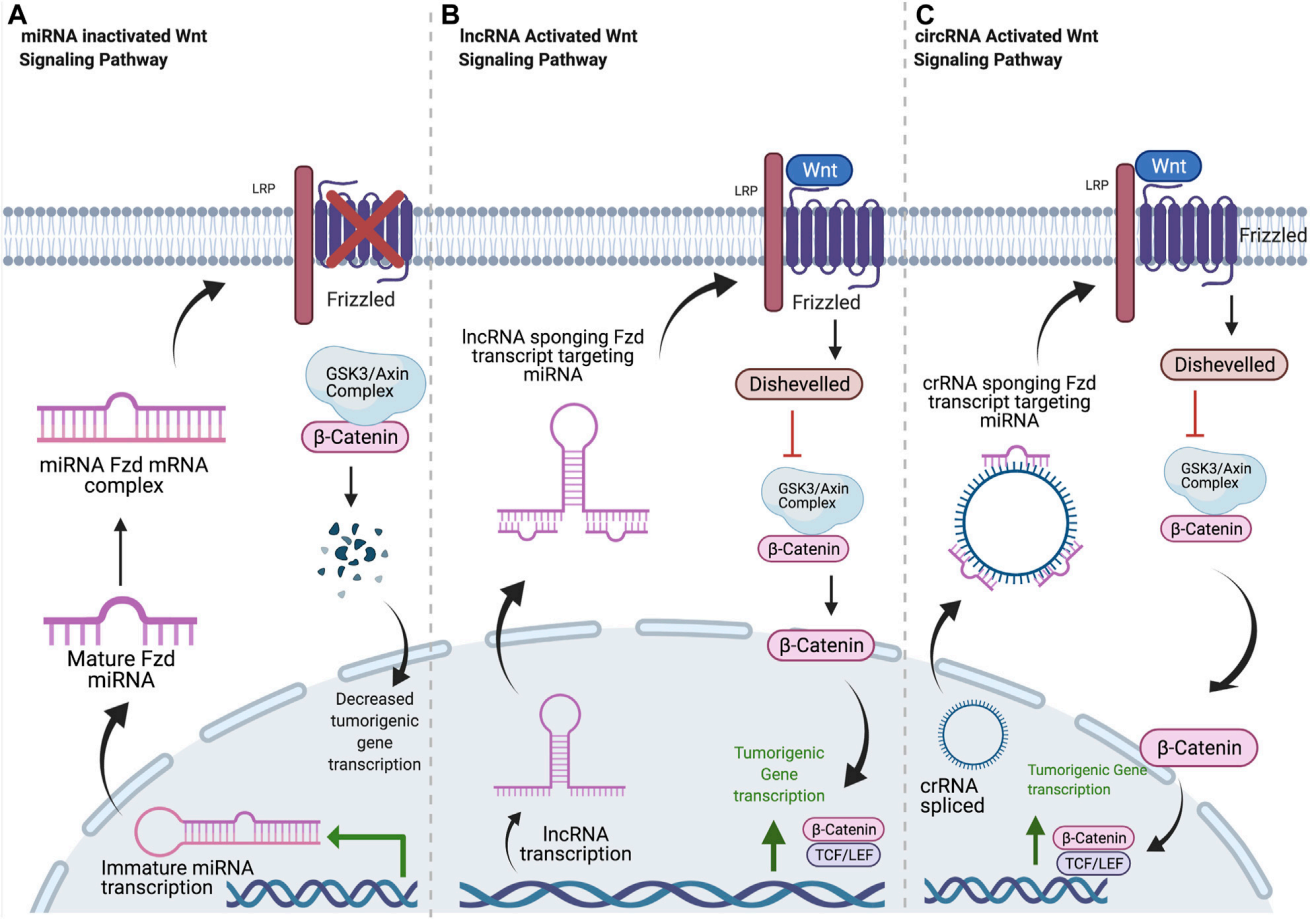
Common mechanisms of ncRNA regulation of Frizzled receptors. **(A)** Mature miRNA oligonucleotides target Fzd transcripts, blocking translation. Decreased Fzd at the plasma membrane results in decreased β-catenin localization to the nucleus and decreased transcription of tumorigenic signaling components. **(B)** Once in the cytosol, lncRNA transcripts sequester miRNA oligos with complementary sequences. This allows for normal Fzd protein expression, leading to increased β-Catenin signaling and tumorigenicity. **(C)** circRNA is spliced and released into cytosol. circRNA sponges miRNA oligos with complementary sequences, resulting in increased Fzd expression and increased tumorigenic signaling through β-catenin. Created with BioRender.com.

MicroRNAs interact with frizzled mRNA transcripts, influencing frizzed protein expression and cancer signaling in various tissues (Figure 1). Fzd1 is moderately expressed in most tissues; however, it is highly expressed in lung, placenta, smooth intestine, and bone tissues. Fzd1 has also been observed to play a role in tumorigenesis in breast cancer, gastric cancer, and non-small cell lung cancer (NSCLC). In an effort to decrease Fzd1 expression and its downstream signaling, several miRNAs have been identified that inhibit Fzd1 mRNA transcription (Su et al., 2016; Cheng et al., 2017a; Sun et al., 2020). For example, miR-135a and miR-8052 act as tumor suppressors in gastric cancer by inhibiting Fzd1 expression, EMT, and cell migration (Cheng et al., 2017a; Sun et al., 2020). It has also been observed that increasing miR-135b expression in chemoresistant NSCLC cell lines decreases Fzd1 expression and increases drug sensitivity (Su et al., 2016). Fzd2, commonly expressed in fetal lung, brain, and kidney, as well as adult cardiac tissue, has been identified as an upregulated tumorigenic receptor in multiple cancers from primary tumors to metastatic tissues (Sagara et al., 1998; Zhang et al., 2015; Fu et al., 2020). MicroRNAs that inhibit Fzd2 expression and signaling lead to inhibition of tumorigenesis. While the mechanism is still unknown, upregulation of CD82 not only decreases c-Met signaling but also upregulates miR-203 expression. Upregulation of miR-203 suppresses both Fzd2 mRNA levels and protein expression in NSCLC cells *in vitro* (Mine et al., 2015). miRNAs miR-17-5p, miR-30a-5p, miR-30a-3p, and miR-34a also decrease Fzd2 expression and inhibit tumorigenesis in cervical carcinoma, esophageal squamous cell carcinoma, and breast cancer, respectively (Qi et al., 2017; Xu et al., 2019a; Bonetti et al., 2020). miR-17-5p also increases drug sensitivity to cervical carcinoma cells treated with Cas-II-gly by inhibiting both Fzd2 and the lncRNA MALAT1 (Xu et al., 2019a). Fzd3 expression has been observed in the adult skeletal muscle, kidney, pancreas, cerebellum, and cerebral cortex tissues, and it is associated with esophageal carcinoma, lung squamous cell carcinoma, leukemia, myeloma, lymphoma, and Ewing sarcoma (Wong et al., 2013). However, microRNAs associated with Fzd3 have only been identified in leukemias thus far. miR-607 downregulates Fzd3 expression, leading to inhibited Wnt/β-catenin signaling, which decreases chronic lymphocytic leukemia (CLL) progression and induces apoptosis (Kirikoshi et al., 2000; Xia et al., 2018). miR-155 and miR-192 inhibit Fzd3/Wnt/β-catenin signaling in acute myeloid leukemia (AML) and decrease transformation, proliferation, and differentiation of AML progenitor cells (Zhang et al., 2017).

Fzd4 expression has a moderate baseline expression in most tissues, with high expression observed in digestive and female reproductive tissues (Uhlen et al., 2015; Protein Atlas (2021). Hum, 2021). Fzd4 has exhibited tumorigenic properties in studies of bladder, prostate, glioma, lung, liver, pancreatic, and cervical carcinoma by supporting proliferation, tumor progression, and metastasis through upregulation of canonical Wnt signaling (Zeng et al., 2018). Multiple miRNAs, including miR-515-5p in NSCLC, miR-101, and miR-493 in bladder cancer, and miR-505 in cervical carcinoma, decrease Fzd4 expression accompanied by tumor-suppressive effects (Ueno et al., 2012; Pardo et al., 2016; Ma C et al., 2017; Chen et al., 2019). A biomarker and potential drug target for NSCLC was identified as an miR-SNP (rs713065) within the 3′UTR binding site of the Fzd4 gene that interacts with miR-204, downregulates Fzd4 expression, and is associated with less aggressive NSCLC tumors (Lin et al., 2017). miRNA-Let7 reduces CD24 + 133+, a cellular indication of stemness, by decreasing Fzd4 expression and Wnt/β-catenin signaling in liver cancer (Cai et al., 2017). Meanwhile, miR-377 and miR-3127-5p inhibit EMT *via* Fzd4 downregulation in prostate cancer and NSCLC, respectively (Formosa et al., 2014; Yang et al., 2018). miR-29c is associated with both Fzd4 and Fzd5 in pancreatic cancer. miR-29c decreases expression of Fzd4, Fzd5, and frizzled co-receptor LRP6, inhibiting pancreatic tumor cell migration and stemness (Jiang et al., 2015).

Fzd5 is normally highly expressed in fetal liver and adult pancreas tissues and moderately expressed in fetal kidney and adult liver tissues (Saitoh et al., 2001a). Fzd5 is associated with breast, pancreatic, gastric, and liver cancer (Zeng et al., 2018). Only two miRNA molecules have been found so far to manipulate Fzd5 expression in cancer. miR-224-5p is associated with FTH1P3 and Fzd5 in oral squamous cell carcinoma (OSCC). FTH1P3 normally acts as a molecular sponge, inhibiting miR-224-5p activity and upregulating Fzd5 expression. However, upregulation of miR-224-5p interferes with this to decrease Fzd5 protein expression, inhibiting colony formation and proliferation (Zhang, 2017). miR-1324 is involved in the circ_0067934/Fzd5 axis in hepatocellular carcinoma (HCC). Normally, circ_0067934 indirectly upregulates Fzd5 by decreasing miR-1324 expression. However, when miR-1324 levels are increased, decreased proliferation, migration, and invasion occur through inhibition of Fzd5/Wnt/β-catenin signaling (Zhu et al., 2018). Fzd6 is moderately expressed in most adult tissues; however, it is highly expressed in endocrine tissues such as the thyroid and adrenal glands (Uhlen et al., 2015; Protein Atlas (2021). Hum, 2021). Fzd6 is connected with many cancer types, including breast, liver, prostate, colorectal, and lung cancers and leukemia (Corda and Sala, 2017). MicroRNAs targeting Fzd6 have mostly been described as tumor-suppressive; however, Fzd6 has been shown to be both upregulated and downregulated in various cancers. In gastric cancer, miR-21 was shown to directly target and inhibit the expression of Fzd6. Interestingly, the downregulation of Fzd6 was shown to increase cell proliferation and migration (Yan et al., 2016). Conversely, in colorectal cancer, Fzd6 expression is upregulated but can be downregulated by miR-199a-5p (Kim et al., 2015). In glioblastoma, two miRNAs, miR-125b and miR-20b, are differentially regulated in proneural and mesenchymal glioblastomas and are inversely expressed compared to Fzd6 expression (Huang et al., 2016). Fzd6 and miR-302b are inversely expressed in OSCC, where inhibition of Fzd6 by miR-302b suppresses metastasis (Sun et al., 2021).

Fzd7 is moderately expressed in most adult tissues also but exhibits high expression levels in neural and reproductive tissues. Fzd7 is essential for the induction of the neural crest and maintaining intestinal homeostasis throughout adulthood (Phesse et al., 2016). zd7 is associated with hepatocellular carcinoma, breast cancer, melanoma, and gastric cancer (Zeng et al., 2018). Regulation of Fzd7 by miRNA occurs in various cancers. miR-199a, miR-542-3p, miR-504, and miR-27b all inhibit tumorigenesis by directly downregulating Fzd7 expression in HCC (Chen et al., 2013; Song et al., 2014; Wu et al., 2017a; Quan et al., 2018). In glioblastoma, miR-144-3p binds to the 3′UTR of Fzd7 and inhibits proliferation, invasion, and migration (Cheng et al., 2017b). Also, in glioblastoma, Fzd7 is expressed inversely to miR-504 (Liu et al., 2019a). In gastric cancer, Fzd7 induces a tumorigenic phenotype in response to *H. pylori* that is suppressed by miR-27b (Geng et al., 2016). Increased expression of miR-944 attenuated doxorubicin resistance in colon cancer by targeting Fzd7 (Xi et al., 2021). A genome-wide screen for miR-23b revealed that it may be a direct modulator of CRC metastasis by regulating Fzd7 (Zhang et al., 2011). Inhibition of Fzd7 by miR-485-5p in melanoma cells was observed with 3′UTR luciferase assays and qPCR analysis (Wu et al., 2017b). In cervical cancer cell lines, miR-142-3p expression is decreased, while Fzd7 was decreased when cells were transfected with miR-142-3p mimics (Deng et al., 2015). miR-613 is predicted to bind the Fzd7 3′UTR and decreases Fzd7 expression when transfected into renal cell carcinoma cell lines (Song et al., 2017). In glioma, patient sample studies were shown to overexpress Fzd7, and both *in vitro and in vivo* interrogation revealed miR-206 targets Fzd7 while also decreasing cell proliferation, migration, and invasion (Zhou et al., 2019). miR-613 has an inverse expressional profile compared to Fzd7 and suppresses cell proliferation and invasion, while overexpression of Fzd7 mitigates this effect (Ren et al., 2016). Last, miR-1 upregulation in breast cancer stem cells directly targets Fzd7 to decrease expression, along with decreasing cell proliferation and invasion (Liu et al., 2015).

Fzd8 is highly expressed in fetal renal and neural tissue, as well as mature renal, pancreatic, cardiac, and skeletal muscle tissue, but has also exhibited tumorigenic influence by supporting cell proliferation, invasion, and metastasis in various cancers (Saitoh et al., 2001b; Li et al., 2017; Yin et al., 2013; Chen et al., 2020). miR-520b and Fzd8 expression is inversely correlated in both osteosarcoma patient samples and cell lines (Wang et al., 2017). In thyroid cancer, miR-370-3p is sponged by other ncRNAs, leading to increased Fzd8 expression (Chen et al., 2018). miR-375 downregulated Fzd8 expression, suppressing metastasis in colorectal cancer (Xu et al., 2016). Finally, miR-99b-5p was shown to directly target Fzd8 in non–small-cell lung cancer, resulting in decreased cell proliferation, invasion, and migration during *in vitro* analysis (Liu et al., 2019b). Fzd9 expression has been identified in mature lung, brain, skeletal muscle, kidney, and male reproductive tissues (Tomizawa et al., 2009). Similar to Fzd6, Fzd9 miRNA regulation contrasts with the other Fzd proteins. Studies of Fzd9 function show it to be upregulated in triple-negative breast cancer, but it is also tumor-suppressive in NSCLC (de Bastos et al., 2021)^,^ (Tennis et al., 2010). However, knowledge of miRNA regulation of Fzd9 is limited, with only one published study showing miR-31 indirectly inhibiting Fzd9 expression and supporting cancer-promoting signaling in NSCLC *in vitro* and *in vivo* (Tennis et al., 2016). Despite studies supporting an oncogenic role for Fzd10, miRNAs that regulate it have yet to be identified. Fzd10 is only normally expressed in fetal tissues and not in adult tissues; however, upregulated Fzd10 protein expression is oncogenic, specifically driving EMT, and is associated with a variety of cancers, including colon, melanoma, and gastric (Wang et al., 2018a; Scavo et al., 2018). Fzd10 is likely targeted by miRNA that will be identified in future studies and offer potential antitumorigenic targets. The extensive current knowledge of miRNA that targets Fzd receptors is summarized in Table 1, with miRNA activity, cancer type, and the frizzled transcript being targeted.

**TABLE 1:** Frizzled receptors and associated miRNA in cancer.

Frizzled	Cancer	miRNA	Activity
Fzd1	Gastric	miR135a	Decreased angiogenesis (Cheng et al., 2017a)
		miR-8052	Decreased EMT, proliferation, and metastasis (Sun et al., 2020)
	NSCLC	miR-135b	Reversed chemoresistance (Su et al., 2016)
Fzd2	ESCC	miR30a-3p	Decreased Wnt/β-catenin pathway Potential biomarker (Qi et al., 2017)
		miR-30a-5p	
	Cervical	miR-17-5p	Increased drug sensitivity (Xu et al., 2019a)
	Breast	miR-34a	Decreased Wnt/β-catenin signaling (Bonetti et al., 2020)
	Lung	miR-203	Cell migration, metastasis, Wnt/β-catenin pathway (Mine et al., 2015)
Fzd3	CLL	miR-607	Induced apoptosis, decreased Wnt/β-catenin signaling (Xia et al., 2018)
	AML	miR-155	Inhibited AML progenitor cell transformation, proliferation, and differentiation (Zhang et al., 2017)
		miR-192	
Fzd4	NSCLC	miR-3127-5p	Decreased EMT *via* Wnt/β-catenin inhibition (Yang et al., 2018)
		miR-204	Targeted a Fzd4/miR/SNP loci to downregulate Fzd4 expression. Associated with increased survival in early stages (Lin et al., 2017)
		miR-515-5p	Decreased migration and invasion (Pardo et al., 2016)
	Cervical	miR-505	Decreased cell proliferation and invasion (Ma C et al., 2017)
	Pancreatic	miR-29c	Suppressed Fzd4 and LRP6 expression to inhibit Wnt/β-catenin signaling (Jiang et al., 2015)
	Bladder	miR-101	Decreased cell migration and metastasis (Chen et al., 2019)
		miR-493	
	Liver	miR-Let7	Decreased stemness of liver cancer cells (Cai et al., 2017)
	Prostate	miR-377	Decreased EMT *via* Wnt/β-catenin inhibition (Formosa et al., 2014)
Fzd5	OSCC	miR-224-5p	Inhibited cell proliferation and colony formation (Zhang, 2017)
	HCC	miR-29c	Suppressed Fzd4 and LRP6 expression to inhibit Wnt/β-catenin signaling (Jiang et al., 2015)
	Pancreatic	miR-1324	Decreased proliferation, migration, and invasion *via* Wnt/β-catenin signaling inhibition (Zhu et al., 2018)
Fzd6	Colorectal	miR-199a-5p	Decreased invasiveness *via* non-canonical WNT signaling (Kim et al., 2015)
	Glioblastoma	miR-20b	Altered regulation of mesenchymal phenotype through STAT3 and NF-kB (Huang et al., 2016)
		miR-125b	
	OSCC	miR-302b	Decreased metastasis (Sun et al., 2021)
Fzd7	HCC	miR-199a	Decreased cell proliferation and survival (Song et al., 2014)
		miR-542-3p	Decreased cell growth (Wu et al., 2017a)
		miR-504	Supported tumor-suppressive phenotype (Quan et al., 2018)
		miR-485-5p	Decreased tumor progression (Wang et al., 2018a)
	Melanoma	miR-485-5p	Decreased cell invasion and proliferation (Wu et al., 2017b)
	CRC	miR-944	Decreased doxorubicin resistance and tumor progression (Xi et al., 2021)
	Colon	miR-23b	Screened as potential inhibitor of metastasis (Zhang et al., 2011)
	Glioblastoma	miR-144-3p	Tumor suppressor and predictive marker for prognosis (Cheng et al., 2017b)
		miR-504	Suppressed EMT signaling (Liu et al., 2019a)
	Glioma	miR-206	Decreased cell proliferation, migration, and invasion (Zhou et al., 2019)
	Cervical	miR-142-3p	Decreased cell proliferation and invasion (Deng et al., 2015)
	RCC	miR-613	Decreased cell proliferation and invasion (Song et al., 2017)
	Prostate	miR-613	Decreased cell proliferation and invasion (Ren et al., 2016)
	Breast	miR-1	Inhibited cell proliferation and migration of breast stem cells (Liu et al., 2015)
	Gastric	miR-27b	Reduced MDR1/P-glycoprotein and β-catenin expression, reduced chemoresistance (Geng et al., 2016)
Fzd8	Colorectal	miR-375	Suppressed metastasis (Xu et al., 2016)
	Spinal osteosarcoma	miR-520b	Decreased cell proliferation, migration, and invasion (Wang et al., 2017)
	Thyroid	miR-370-3p	Decreased cell proliferation and invasion (Chen et al., 2018)
Fzd9	NSCLC	miR-31	Decreased EMT signaling (Tennis et al., 2016)

Fzd10 was not included in the table as no miRNAs have been identified. NSCLC, non–small-cell lung cancer; ESCC, esophageal cancer; CLL, chronic lymphocytic leukemia; AML, acute myeloid leukemia; OSCC, oral squamous cell carcinoma; HCC, hepatocellular carcinoma; CRC, colorectal cancer; RCC, renal cell carcinoma.

## Long Non-Coding RNA

The therapeutic potential of targeting lncRNAs was introduced in 2009; however, research regarding their influence on tumorigenic potential related to Fzds has been limited until recently (Ponting et al., 2009). The influence of lncRNA on Fzd protein expression and related downstream signaling and tumor progression is mostly a result of their ability to sequester miRNA transcripts as a competing endogenous RNA (ceRNA) molecule (Figure 1). LEF1-AS1 acts as a ceRNA against miR-328 by upregulating CD44. This resulted in an increase in Fzd2/Wnt/β-catenin signaling by recruiting C-myc to upregulate Fzd2 transcription in prostate cancer (Li et al., 2020b). By *in silico* and *in vitro* analysis, MALAT1 influenced Fzd2 expression by competing for miR-17-5p (Xu et al., 2019a). SNHG10 upregulated Fzd3 expression in osteosarcoma, both *in vitro* and *in vivo*, by binding miR-182-5p and increasing Wnt/β-catenin signaling and tumorigenesis (Zhu et al., 2020). DLX6-AS1 binds miR-497-5p *in vitro* and increases Fzd4 expression in pancreatic cancer, leading to increased expression of EMT markers (Yang et al., 2019). Upregulation of HOXD-AS1 expression in ovarian cancer (OC) competitively binds miR-608, an inhibitory miRNA of Fzd4, *in vitro* and *in vivo*, leading to high Fzd4 expression that correlates with ovarian cancer cell migration, invasion, and proliferation (Wang et al., 2018b). Fzd5 expression in oral squamous cell carcinoma (OSCC) is increased by FTH1-3, which acts as a ceRNA against miR-224-5p to remove repression of Fzd5 expression and induce proliferation and colony formation in OSCC cells (Zhang, 2017). Both DLX6-AS1 and CASC9 sponge miRNA miR-497-5p, which increases Fzd6 expression, along with tumor growth and metastasis in pancreatic cancer and bladder cancer, respectively (Yang et al., 2019; Zhan et al., 2020). In hepatocellular carcinoma, DSCR8 increases Fzd7 expression by sequestering miR-485-5p (Wang et al., 2018a). In bladder cancer, ROR1-AS1 acts as a ceRNA for miR-504 to increase Fzd7 expression (Chen and Fu, 2020). Increased expression of Fzd7 leads to increased tumorigenesis in both cancer types. In triple negative breast cancer, AWPPH is expression is positively correlated with Fzd7, and it may act as a ceRNA for an unidentified miRNA (Wang et al., 2018c).

Alternatives to the ceRNA mechanism have been described for lncRNA, although many studies have yet to identify mechanisms for observed lncRNA effects on targets. VIM antisense RNA 1 (VIM-AS1) expression enhanced tumorigenic pathways by downregulating miR-8052 expression and increasing expression of Fzd1 in human gastric cancer (GC) tissue (Sun et al., 2020). In a microarray of human blood and tissue samples, NR_110882 promoted tumorigenesis in colorectal cancer (CRC) by increasing Fzd2 expression, Wnt2/Fzd2 binding, and Wnt/β-catenin signaling (Tian et al., 2019). GATA6-AS1 induces hypermethylation of the Fzd4 promoter region *via* EZH2 recruitment in gastric cancer, inhibiting Fzd4 expression and reversing EMT (Li et al., 2020a). AK126698 regulates Fzd8 similarly to miRNA, where it decreases Fzd8 expression by directly binding the mRNA transcript, leading to reduced protein expression, cell proliferation, and migration in NSCLC (Fu et al., 2016). While lncRNA regulation has yet to be described for both Fzd9 and Fzd10, the potential for investigation is clear as they have been shown to be differentially expressed during tumorigenesis in various cancers (Nagayama et al., 2009; Gong et al., 2014; Tennis et al., 2016). LncRNA that affect Fzds may be therapeutic targets by interfering with their ceRNA activity to restore normal Fzd expression or by manipulating other mechanisms of lncRNA activity.

## Circular RNA

CircRNA generally leads to upregulated Fzd expression and downstream signaling through various circRNA/miRNA/Fzd pathways. All of the circRNAs associated with Fzds to date have oncogenic effects by sequestering tumor-suppressive miRNAs (Figure 1). In chronic lymphocytic leukemia (CLL) cells, circ-CBFB indirectly upregulates Fzd3 expression and downstream Wnt/β-catenin signaling by inhibiting miR-607 (Xia et al., 2018). Similar mechanisms were observed in CRC spheroid cells where circ_0082096 and circ_006631 upregulate Fzd3 expression by inhibiting miR382, miR-579, miR-224, and miR-548c (Rengganaten et al., 2020). CircRNA_100290 also influences colorectal carcinoma (CRC) by inhibiting miR-516b in CRC cells, indirectly upregulating Fzd4 and the Wnt/β-catenin pathway (Fang et al., 2018). With numerous miRNAs already associated with Fzd4, there is strong potential for further investigation into circRNA that influences Fzd4 signaling in cancer. Meanwhile, circ_0067934 is upregulated in hepatocellular carcinoma (HCC) tissues, and knocking it down significantly reduced cell proliferation, migration, and invasion, while also drastically increasing apoptosis rates *in vitro* (Zhu et al., 2018). Circ_0067934 inhibits miR-1324, an miRNA that decreases Fzd5 expression and downstream Wnt/β-catenin signaling in pancreatic cancer (Zhu et al., 2018). A similar mechanism was observed in gastric cancer as circ_MTHFD2 sponges miR-124, leading to an increase in Fzd5 protein expression and contributing to multidrug resistance (Xu et al., 2019b). In HCC, increased Fzd7 expression is observed when miR-485-5p is sponged by DSCR8 (Wang et al., 2018a). Fzd7 expression is increased in CRC due to circ_CSPP1 sponging miR-944 and in glioma due to circ_0000177 sequestering miR-638. Both circ_CSPP1 and circ_0000177 expression increased cell proliferation and invasion in these cancers (An et al., 2018; Xi et al., 2021). Fzd8 and NEK6 are overexpressed in thyroid cancer, where NEK6 is a circRNA that targets miR-370-3p, which targets Fzd8 3′UTR (Chen et al., 2018). Fzd1, Fzd2, Fzd6, Fzd9, and Fzd10 currently do not have any circRNA molecules associated with regulation of their expression, despite their obvious role in cancer signaling and tumor progression, highlighting the potential for future research.

## Discussion

While studies are still limited, the importance of non-coding RNA in Fzd receptor regulation is clearly emerging. Ongoing research will contribute to improved understanding of the mechanisms of individual Fzd regulation in specific tissue and cellular contexts. Manipulating Fzd expression or activity in cancer cells could offer new approaches to prevention and therapy. Small-molecule drugs could target Fzd-binding pockets, Wnt ligands, co-receptors, or heterotrimeric G_i_ proteins to control downstream intracellular signaling. Targeting non-coding RNAs present an additional approach to inhibiting oncogenic pathways or enhancing tumor-suppressive pathways. Challenges of targeting Fzds include lack of high-resolution crystal structures for Fzds and context-dependent associations of individual Wnts and Fzds (Schulte and Kozielewicz, 2020; Zhao et al., 2020). Using non-coding RNA to target Fzd activity could circumvent some of these challenges by altering expression, instead of attempting to bind to Fzd receptors or by offering tissue- or disease-specific targeting. The larger volume of studies on miRNA and Fzds points to miRNA as the first potential approach for using non-coding RNA to target Fzds in cancer. This is supported by their demonstrated ability to downregulate oncogenic Fzd expression, inducing antitumorigenic effects both *in vitro* and *in vivo.* miRNAs have also been used as biomarkers for tumor prognosis and susceptibility to drug resistance, increasing their appeal as potential mechanistic targets that can be measured as markers of response (Lan et al., 2015; Hanna et al., 2019).

ncRNA molecules have therapeutic potential through regulation of Fzd-initiated oncogenic cell signaling pathways; however, the study of ncRNA in clinical cancer settings has been thus far limited to miRNA. There are clinical trials investigating miRNAs as biomarkers for diagnosis, drug sensitivity, or therapeutic agents in cardiac disease, cancer, neurodegenerative disorders, and viral infections. For example, miR199a-5p and miR-126-3p were found to influence endothelial dysfunction in Fabry disease (FD) and can be used as a diagnostic factor to identify individuals with FD (Cammarata et al., 2018). Similarly, miR-30e was identified as a diagnostic marker in schizophrenic patients following peripheral plasma and peripheral blood mononuclear cell analysis (Sun et al., 2015). Approaches to targeting miRNAs include modified siRNAs, anti-sense oligonucleotides, and small molecules (Wang et al., 2019). miRNA inhibitors and mimics have been administered by intravenous or intratumoral injections in clinical trials. This method of delivery presents obstacles such as off-target effects, poor target reach, low stability, and short circulation time (Hanna et al., 2019; Lee et al., 2019). Nanoparticles represent a potentially improved vector to deliver miRNA drugs that provides increased stability, circulation time, and target accuracy (Lee et al., 2019). This approach was supported by a study that observed a decrease in breast cancer metastasis, following delivery of an miR-708 mimic *via* nanoparticles (Ramchandani et al., 2019). lncRNA can be targeted by several approaches, including anti-sense oligonucleotides, or CRISPR, although clinical utility of these approaches for cancer is still under study. Advances in *ex vivo* and *in vivo* modeling, such as large animals or tissue slice models, are helping to address pre-clinically the safety and efficacy issues that often halt ncRNA drugs early in the development pipeline. A recently investigated alternative, small-molecule inhibitors of lnRNA, may offer a non-sequence targeting option with easier delivery modalities (Chen et al., 2021). With so much still to be learned about lncRNA function and interactions with Fzd, it is likely this approach will not be seen in clinical application for some time. Approaches to modulating circRNAs have been similar to lncRNAs, and they also have potential as therapeutic tools due to their stability and ability to be engineered with multiple binding sites (Rajappa et al., 2020). However, even more so than lncRNA, despite intriguing initial results on the role of circRNA in cancer, considerable additional effort is required to advance circRNA therapy to the clinic. miRNA is currently the most promising ncRNA target in clinical trials, but new therapeutic models will certainly emerge as knowledge and techniques for studying lncRNA and circRNA improve and increase. As research on Fzd receptors and non-coding RNA continues to grow, corresponding investigation of the potential to target Fzd receptor activity through non-coding RNAs will enhance the development of Fzd-based biomarkers, chemoprevention, and chemotherapy.
